# Supplementary material for: Influence of arm swing on cost of transport during walking
Source: Biol Open. 2019 May 29;8(6):bio039263. doi: 10.1242/bio.039263 (PMC6602321; doi:10.1242/bio.039263)
Supplement: Supplementary information [file biolopen-8-039263-s1.pdf]

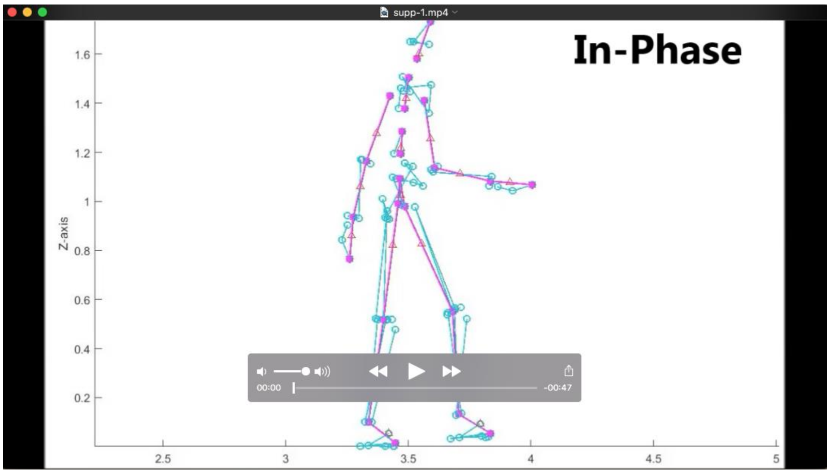

Movie 1. The seven conditions investigated in this study. Displayed are several seconds of each condition as measured with the Xsens system, after data analysis. The conditions are shown in the following order: Inphase, Passive, Active, Normal, Extra 1, Extra 2 and Extra 3.
